# The Self-Bleaching Process of Microcystis aeruginosa is Delayed by a Symbiotic Bacterium Pseudomonas sp. MAE1-K and Promoted by Methionine Deficiency

**DOI:** 10.1128/spectrum.01814-22

**Published:** 2022-06-30

**Authors:** Jaejoon Jung, Ju Hye Baek, Yunho Lee, Sang Eun Jeong, Che Ok Jeon

**Affiliations:** a Department of Life Science, Chung-Ang University, Seoul, Republic of Korea; b Department of Food Science and Biotechnology, CHA University, Pocheon, Gyeonggi-do, Republic of Korea; c Nakdonggang National Institute of Biological Resources, Sangju-si, Gyeongsangbuk-do, Republic of Korea; University of Manitoba

**Keywords:** *Microcystis aeruginosa*, bleaching, methionine deficiency, *Pseudomonas*, *metZ* mutant, bleaching compounds

## Abstract

Various interactions between marine cyanobacteria and heterotrophic bacteria have been known, but the symbiotic relationships between *Microcystis* and heterotrophic bacteria remain unclear. An axenic *M. aeruginosa* culture (NIES-298) was quickly bleached after exponential growth, whereas a xenic *M. aeruginosa* culture (KW) showed a normal growth curve, suggesting that some symbiotic bacteria may delay this bleaching. The bleaching process of *M. aeruginosa* was distinguished from the phenomena of previously proposed chlorosis and programmed cell death in various characteristics. Bleached cultures of NIES-298 quickly bleached actively growing *M. aeruginosa* cultures, suggesting that *M. aeruginosa* itself produces bleach-causing compounds. Pseudomonas sp. MAE1-K delaying the bleaching of NIES-298 cultures was isolated from the KW culture. Bleached cultures of NIES-298 treated with strain MAE1-K lost their bleaching ability, suggesting that strain MAE1-K rescues *M. aeruginosa* from bleaching via inactivation of bleaching compounds. From Tn5 transposon mutant screening, a *metZ* mutant of strain MAE1-K (F-D3) unable to synthesize methionine, promoting the bleaching of NIES-298 cultures but capable of inactivating bleaching compounds, was obtained. The bleaching process of NIES-298 cultures was promoted with the coculture of mutant F-D3 and delayed by methionine supplementation, suggesting that the bleaching process of *M. aeruginosa* is promoted by methionine deficiency.

**IMPORTANCE** Cyanobacterial blooms in freshwaters represent serious global concerns for the ecosystem and human health. In this study, we found that one of the major species in cyanobacterial blooms, Microcystis aeruginosa, was quickly collapsed after exponential growth by producing self-bleaching compounds and that a symbiotic bacterium, Pseudomonas sp. MAE1-K delayed the bleaching process via the inactivation of bleaching compounds. In addition, we found that a *metZ* mutant of strain MAE1-K (F-D3) causing methionine deficiency promoted the bleaching process of *M. aeruginosa*, suggesting that methionine deficiency may induce the production of bleaching compounds. These results will provide insights into the symbiotic relationships between *M. aeruginosa* and heterotrophic bacteria that will contribute to developing novel strategies to control cyanobacterial blooms.

## INTRODUCTION

Cyanobacteria, the oldest oxygenic photosynthetic organism, is the fundamental primary producer together with eukaryotic algae in aquatic environments and is a major contributor to the global carbon, sulfur, and nitrogen cycles (1, 2). Although cyanobacteria also belong to prokaryotic bacteria like heterotrophic bacteria, owing to their photosynthetic characteristics, they can coexist with diverse heterotrophic bacteria using a variety of interactions, like eukaryotic algae (3–5). Cyanobacteria primarily provide heterotrophic bacteria with carbon sources, and heterotrophic bacteria benefit the growth of cyanobacteria through various interactions (6, 7). To date, interactions between oligotrophic marine cyanobacteria such as *Synechococcus* and *Prochlorococcus* and heterotrophic bacteria have been extensively studied (5, 8).

Cyanobacterial blooms are a global concern because they cause various detrimental problems in aquatic ecosystems (9). Microcystis aeruginosa, one of the major species in freshwater algal blooms, is a dangerous cyanobacterium because it produces toxic metabolites such as microcystin and microviridin, which are lethal to wildlife and humans (10–13). Moreover, *M. aeruginosa* is found in a wide range of habitats, including temperate and tropical lakes, rivers, and even brackish waters, indicating their excellent adaptability to various environmental conditions (14–16). It has been suggested that symbiotic heterotrophic bacteria may also be involved in the survival or adaption of *M. aeruginosa* (17, 18). However, the interactions between *M. aeruginosa* and heterotrophic bacteria are not well known.

When exposed to unfavorable conditions, cyanobacteria increase their survival chances through a variety of responses (11, 19). *M. aeruginosa* also have various survival strategies in response to environmental changes, and their successful proliferation or stressful response may be attributed to symbiotic heterotrophic bacteria. To address these questions, in this study, we compared the growth profiles of axenic and xenic *M. aeruginosa* cultures and found that the axenic culture was quickly bleached to death after the exponential growth phase. Additionally, we observed that the bleaching process of *M. aeruginosa* is different from its survival or adaption strategies and a symbiotic heterotrophic bacterium delays the bleaching process of *M. aeruginosa*, which provides novel insights into the relationships between *M. aeruginosa* and the heterotrophic bacteria.

## RESULTS

### Cell death by bleaching in axenic *M. aeruginosa* culture was fostered.

**FIG 1:**
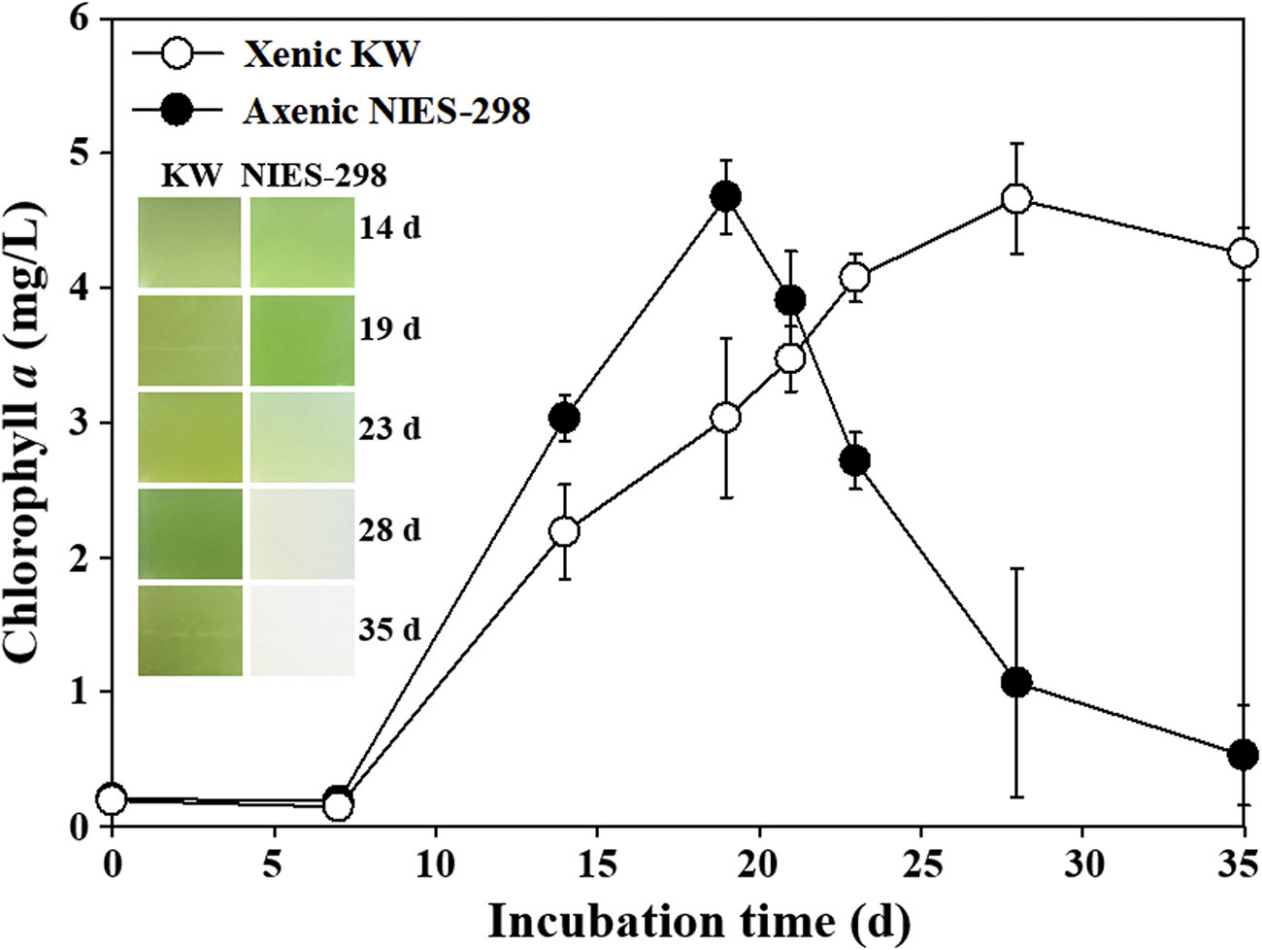
Growth profiles of xenic (KW) and axenic (NIES-298) cultures of *M. aeruginosa*. Images showing colors of the xenic and axenic cultures during growth are displayed as insets inside the figure.

To investigate the effects of symbiotic microbes on the growth of *M. aeruginosa*, the growths of an axenic culture, NIES-298, and a xenic culture, KW, were compared (Fig. 1). The xenic culture, KW, displayed a typical cell growth profile having lag, exponential, stationary, and death phases, whereas the axenic culture, NIES-298, was quickly bleached (to white) without a stationary phase after reaching the maximum growth (at 19 days [d]). The subculture (2%) of bleached NIES-298 culture during the end of death phase (after 28 d) to fresh BG-11 broth did not show any growth, suggesting that the bleached NIES-298 cells are no longer alive. However, the subculture of the KW culture of 28 d to fresh BG-11 broth showed good growth.

**FIG 2:**
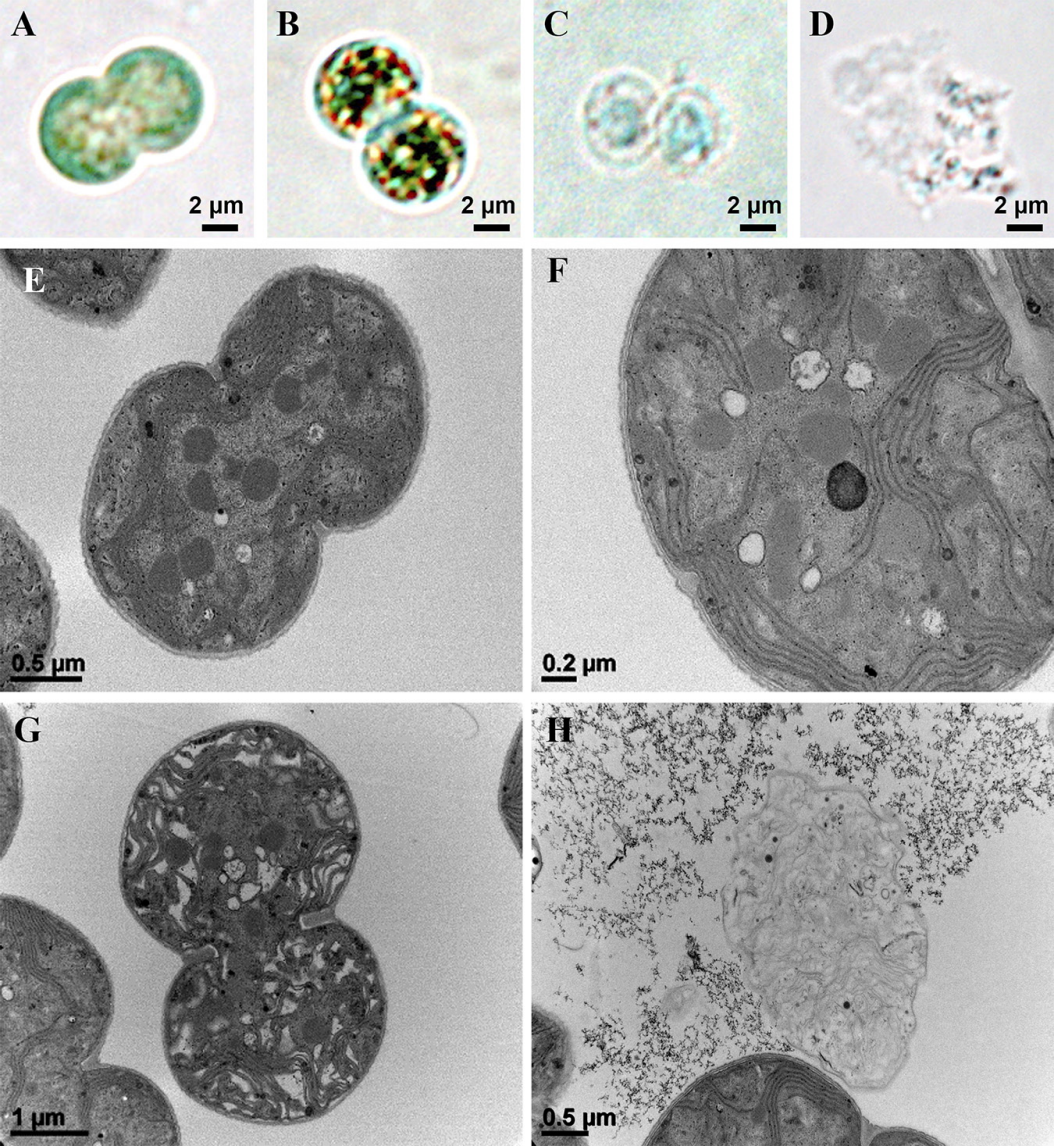
Microscopic analyses showing the general bleaching process of cells in the axenic NIES-298 culture. Differential interference contrast (A–D) and transmission electron (E–H) microscopic images. (A and E) General morphology of cells during the exponential growth phase. (B and F) General morphology of cells during the initial death phase. (C, D, G, and H) General morphology of cells during the late death phase.

Differential interference contrast microscope (DICM) images showed that the xenic KW cells stayed in healthy and green states even after 28 d, whereas the axenic NIES-298 cells were mostly bleached and lysed at 28 d (Fig. S1 in the supplemental material). A DICM was used to investigate the cell-bleaching process in NIES-298 culture and showed that most cells in the exponential growth phase were healthy and evenly green (Fig. 2A), whereas in the death phase they became unevenly green, bleached, and eventually lysed (Fig. 2B to D). Transmission electron microscope (TEM) analysis also showed that most NIES-298 cells in the exponential growth phase had intact cell walls, cell membranes, and intracellular structures (thylakoids and granules) (Fig. 2E), whereas those in the death phase had many void-like and clumped intracellular structures (Fig. 2F and G) and released intracellular matter through cell membrane disruptions (Fig. 2H).

The overall microscopic investigation for the bleaching process of the NIES-298 culture suggested that chlorophyll bleaching and destruction of intracellular structures occurred first, followed by cell membrane breakage and intracellular matter release. Other xenic *M. aeruginosa* cultures, KW, FBC0000003, and NICRCY0000000452, were also eventually bleached and lysed through a similar bleaching process after 40 d, like NIES-298 culture (data not shown).

### *M. aeruginosa* itself produces compounds that cause the self-bleaching process.

To gain clues as to why cells of axenic *M. aeruginosa* cultures were rapidly bleached, several possible causes were investigated. First, to investigate the possibility of a shift from lysogeny to a lytic life cycle of cyanophages in strain NIES-298, the genome of strain NIES-298 was completely sequenced (CP046058–9) and cyanophage-associated genes were bioinformatically analyzed. Many putative cyanophage-associated genes, including phage protein gp37 (*gp37*), tail sheath protein (D3800_09805), prevent-host-death protein (*yhhV*), and phage protein gp11 (*gp11*), were identified (Fig. S2). However, the predicted cyanophage genes were separately distributed into two distant regions of the chromosome, suggesting that the predicted cyanophage genes have low possibility of becoming a lytic cyanophage. Additionally, TEM observation (data not shown) and a viral plaque formation test (Fig. S3) were attempted to detect cyanophages from the bleached NIES-298 culture, but they all failed. These results suggest that lytic cyanophage is not a cause of the bleaching of *M. aeruginosa* cultures.

To investigate the bleaching process of *M. aeruginosa* at the transcriptional level, the transcriptomes of NIES-298 cells at the midexponential (ME), late exponential (LE), and early death (ED) phases were analyzed (SRR17982185–7). The transcriptional expressions of 284 functional genes (6.04% of the total genes) exhibited >2-fold changes at the ED phase compared to the ME or LE phases (Table S1). Among them, genes associated with photosynthesis, carbon fixation, and ATPase were generally downregulated at the ED phase, indicating the decrease of overall photosynthetic activity. However, the transcriptional expressions of oxidative stress defense-associated genes such as SOD (D3800_00390 and D3800_14880), peroxiredoxin (D3800_02530), and peroxidase (D3800_05470) and the cyanophage-related genes did not show significant differences, suggesting that they may not be associated with the bleaching process of *M. aeruginosa*. The treatment of the NIES-298 cultures with SOD, catalase, and glutathione also did not delay the bleaching of NIES-298 cultures (data not shown). Four putative caspase genes (D3800_09480, D3800_10800, D3800_10885, and D3800_19795) probably mediating programmed cell death were identified by BLASTP based on the known caspase amino acid sequences in *M. aeruginosa* PCC 7806, but they were not upregulated at the ED phase (20).

**FIG 3:**
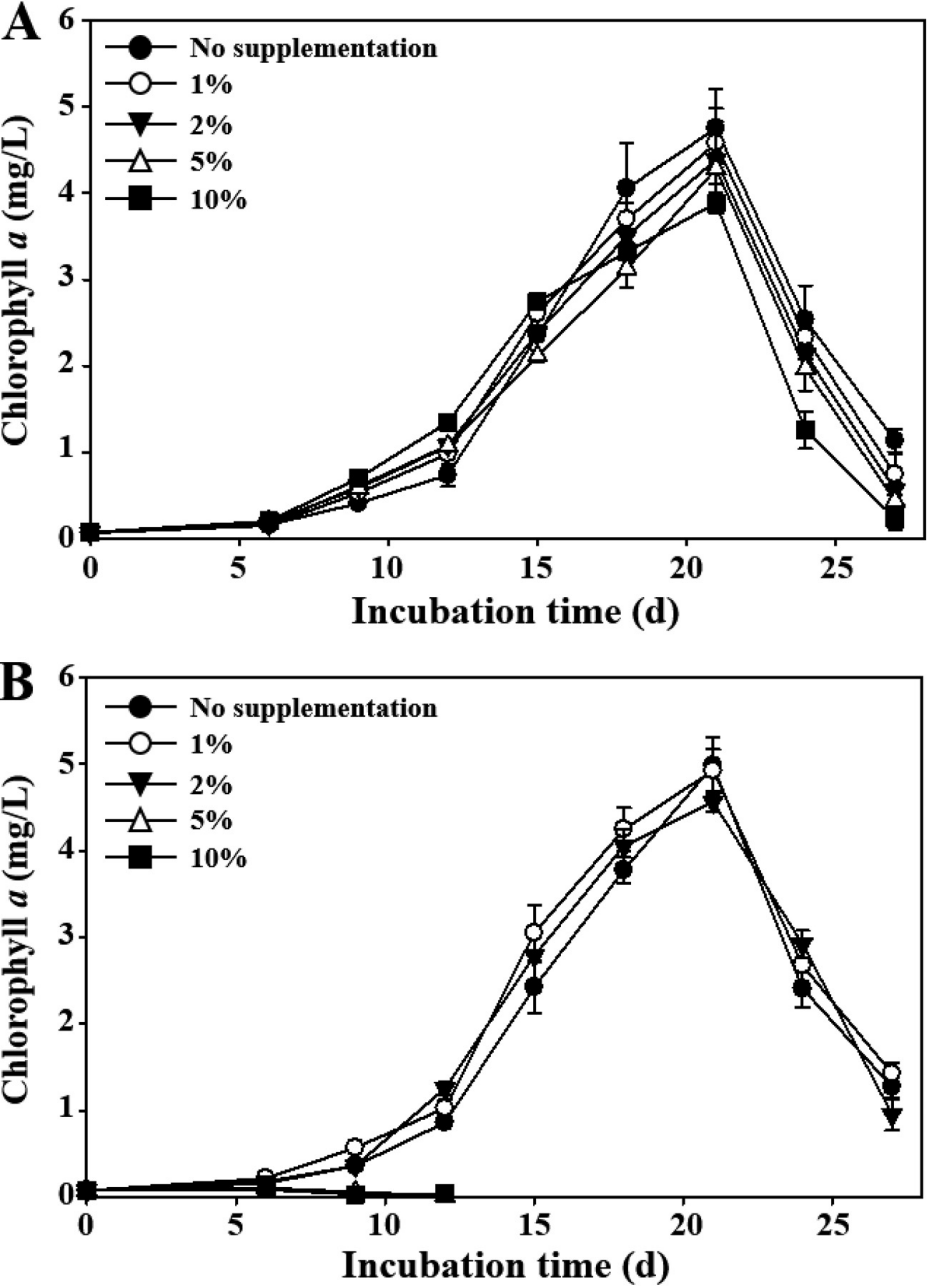
Effects of NIES-298 cultures on the growth of strain NIES-298. The growth of NIES-298 cultures was evaluated in BG11 broth supplemented with different amounts of filtered NIES-298 cultures, which were prepared by filtering NIES-298 cultures at the exponential growth (A) and death (B) phases.

The putative *nblA* (D3800_08230) gene that may be associated with the chlorosis of *M. aeruginosa* occurring under the depletion of nitrogen sources was slightly upregulated at the ED phase. However, it was shown that a nitrogen source, nitrate, was not depleted during the entire cultivation period (Fig. S4), which suggests that the expression of the *nblA* gene in *M. aeruginosa* NIES-298 cultures might not be associated with the chlorosis by nitrogen depletion. The subcultures of NIES-298 cultures during the exponential growth phase (before 18 d in Fig. 1) to fresh BG-11 media were always successful regardless of their transfer sizes. However, during the death phase (after 20 d in Fig. 1), small amounts of transfers (less than 2%) exhibited successful subcultures, whereas large amounts of transfers (approximately 5%) resulted in the growth failure of NIES-298 cultures (data not shown). These results suggested that the bleached cultures of *M. aeruginosa* NIES-298 might contain compounds causing the bleaching process; thus, the effects of bleached cultures on the growth of NIES-298 cultures were tested. The subcultures of NIES-298 cultures to BG-11 broth supplemented with filtered culture solution of the exponential growth phase were always successful regardless of supplementation amounts (Fig. 3A). However, no growth with complete bleaching was observed in the subcultures of NIES-298 culture to BG-11 broth supplemented with more than 5% (vol/vol) filtered bleached cultures of the death phase (Fig. 3B). Particularly, exponentially growing NIES-298 cultures also were shown to be completely bleached by the addition of filtered bleached culture derived from the end of the death phase (data not shown). These results suggest that *M. aeruginosa* itself may produce compounds that cause the self-bleaching process.

### A symbiotic bacterium, Pseudomonas sp. MAE1-K, delayed the bleaching of *M. aeruginosa* through the inactivation of bleaching compounds.

**FIG 4:**
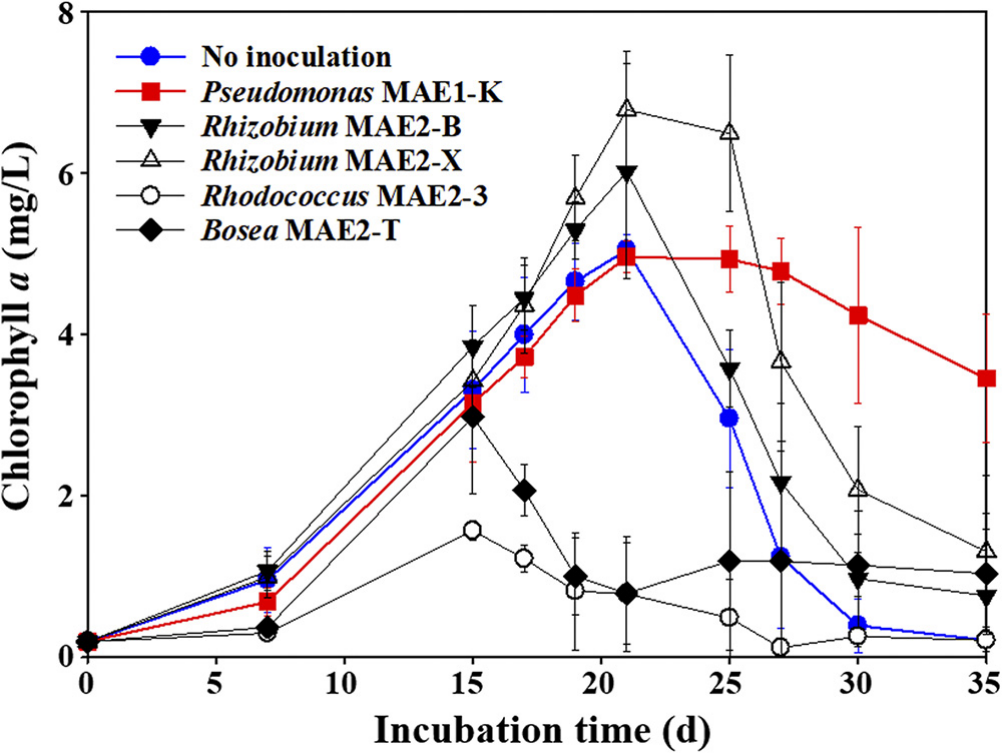
Effects of bacterial strains isolated from the xenic KW culture on the growth of axenic NIES-298 cultures.

From the results above, we hypothesized that some symbiotic microbes may delay the bleaching of *M. aeruginosa* through the inactivation of bleaching compounds. Therefore, we isolated bacterial strains from xenic KW culture (Table S2) and evaluated their effects on the growth of NIES-298 cultures (Fig. 4). *Rhodococcus* and *Bosea* strains (MAE2-J and MAE2-T) that were isolated at low abundances inhibited the growth of NIES-298 cultures. On the contrary, *Rhizobium* strains (MAE2-B and MAE2-X) increased the maximum growth of NIES-298 cultures, but the cultures were also rapidly bleached after their maximum growth, like axenic NIES-298 cultures. However, interestingly, the Pseudomonas strain (MAE1-K) that was most abundantly isolated delayed the bleaching process of NIES-298 cultures, like the growth profile of xenic KW cultures.

**FIG 5:**
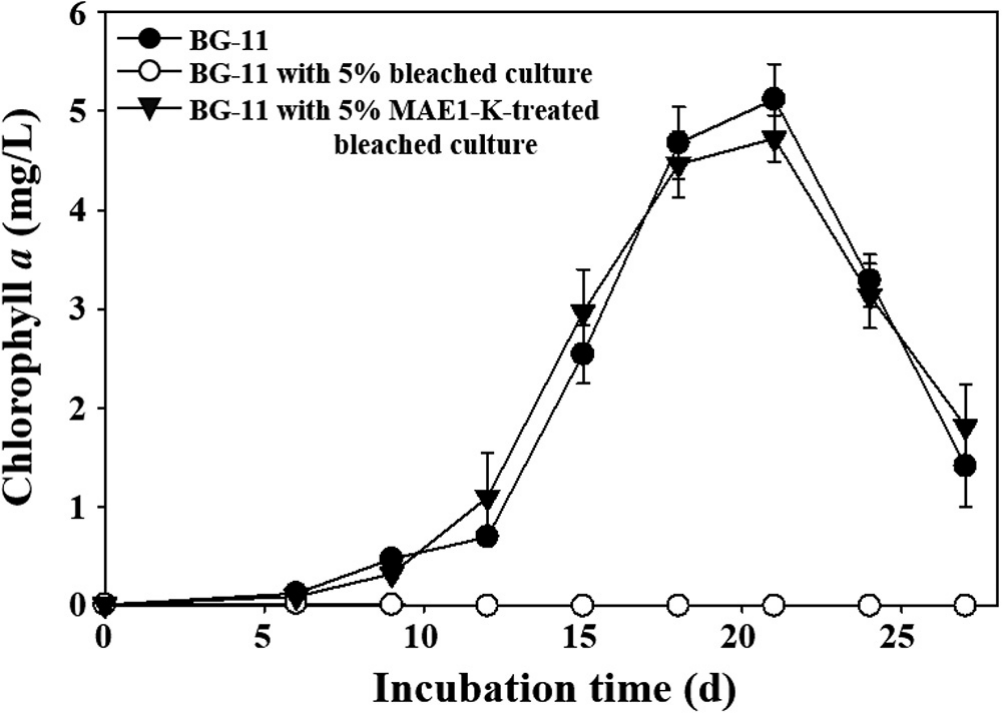
Pseudomonas sp. MAE1-K with the ability to inactivate the bleaching property of bleached culture to NIES-298 culture. The bleached culture was prepared by filtering bleached NIES-298 culture at the end of the death phase. The growth of NIES-298 cultures was evaluated in BG11 broth supplemented with 5% (vol/vol) bleached culture or 5% MAE1-K-treated bleached culture.

The effects of Pseudomonas putida KT2440 and Pseudomonas aeruginosa PAO1 as controls of strain MAE1-K on the growth of strain NIES-298 were also investigated. P. putida KT2440 also delayed the bleaching of NIES-298 culture, like strain MAE1-K, while P. aeruginosa PAO1 inhibited the growth of NIES-298 culture without delayed effect of the bleaching process (data not shown), suggesting that the delay ability of Pseudomonas strains in the bleaching process of *M. aeruginosa* may be strain-specific. The bleaching property of bleached cultures was lost by the treatment of strain MAE1-K (Fig. 5), which suggests that strain MAE1-K may delay the bleaching process of NIES-298 culture by inactivating compounds causing the bleaching process.

### A *metZ* mutant of strain MAE1-K promoted the bleaching process of *M. aeruginosa*.

The genome of strain MAE1-K was completely sequenced (CP023641). Strain MAE1-K has a single circular chromosomal genome of 5.16 Mb with a G+C content of 64.5%, and a total of 4,698 protein-coding genes, 4 rRNA operons (16S, 23S, and 5S), and 65 tRNA genes were predicted (Fig. S5). To identify genes to delay the bleaching of NIES-298 cultures in strain MAE1-K, a Tn5 transposon mutant library of strain MAE1-K was constructed and a total of 1,920 transposon mutants were obtained. The effects of all mutants on the growth of NIES-298 culture were assessed, and a transposon mutant (F-D3) that evidently promoted the bleaching of NIES-298 culture was selected.

**FIG 6:**
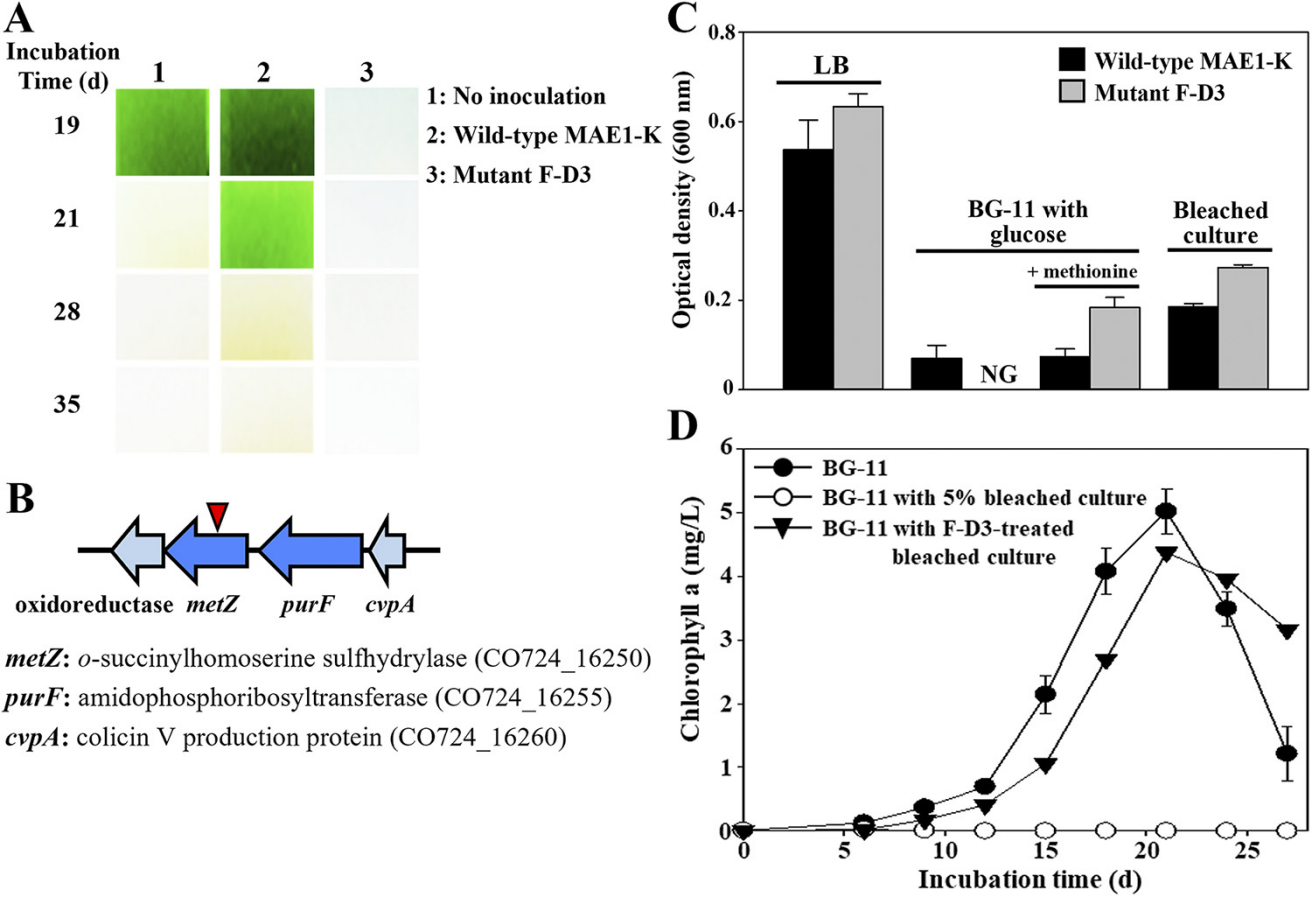
A transposon mutant (F-D3) of strain MAE1-K losing the ability to delay the bleaching of NIES-298 culture. (A) Bleaching images of NIES-298 culture promoted by strain F-D3. Wild-type MAE1-K and mutant F-D3 were inoculated into the NIES-298 cultures of the exponential growth phase (at 15 d). (B) A genetic map indicating the transposon insertion site in mutant F-D3. (C) Growth of wild-type MAE1-K and mutant F-D3 in LB broth, BG-11 with glucose (0.1%) or with glucose and methionine (0.5 mM), and bleached culture. The bleached culture was prepared by filtering bleached NIES-298 culture at the end of the death phase. Optical density (600 nm) was measured after 24 h aerobic incubation (30°C). NG, no growth. (D) Mutant F-D3 that can still inactivate the bleaching property of bleached culture to NIES-298 culture.

The bleaching of NIES-298 cultures occurred rapidly after the inoculation of mutant F-D3 into NIES-298 cultures, indicating that mutant F-D3 completely lost the ability of wild-type MAE1-K to delay the bleaching of NIES-298 cultures and rather evidently promoted the bleaching process of NIES-298 cultures (Fig. 6A). The analysis of the transposon insertion site in mutant F-D3 revealed that the gene encoding *O*-succinylhomoserine sulfhydrylase (*metZ*, CO724_16250) converting *O-*succinyl-l-homoserine to l-homocysteine in the l-methionine biosynthesis was knocked out (Fig. 6B), suggesting that mutant F-D3 lost the ability to synthesize l-methionine. Indeed, mutant F-D3 did not grow in BG11 broth without methionine supplemented with glucose, but it grew well in BG11 broth supplemented with methionine (Fig. 6C), indicating that mutant F-D3 needs methionine for growth. Both wild-type MAE1-K and mutant F-D3 in filtered bleached culture grew well, suggesting that bleached culture contains methionine as well as a carbon source. Mutant F-D3 showed better growth than wild-type MAE1-K in all media containing methionine tested, suggesting that mutant F-D3 may consume nutrients (methionine) faster than wild-type MAE1-K for growth. Interestingly, mutant F-D3 was still shown to have the ability to inactivate the bleaching property for NIES-298 cultures of bleached cultures, like the wild-type MAE1-K (Fig. 6D). These results suggest that the promoted bleaching of NIES-298 cultures by mutant F-D3 may be related to the loss of the ability of strain MAE1-K to synthesize methionine, not the loss of the ability to inactivate compounds causing the bleaching process.

### Methionine deficiency induced the self-bleaching process of *M. aeruginosa*.

**FIG 7:**
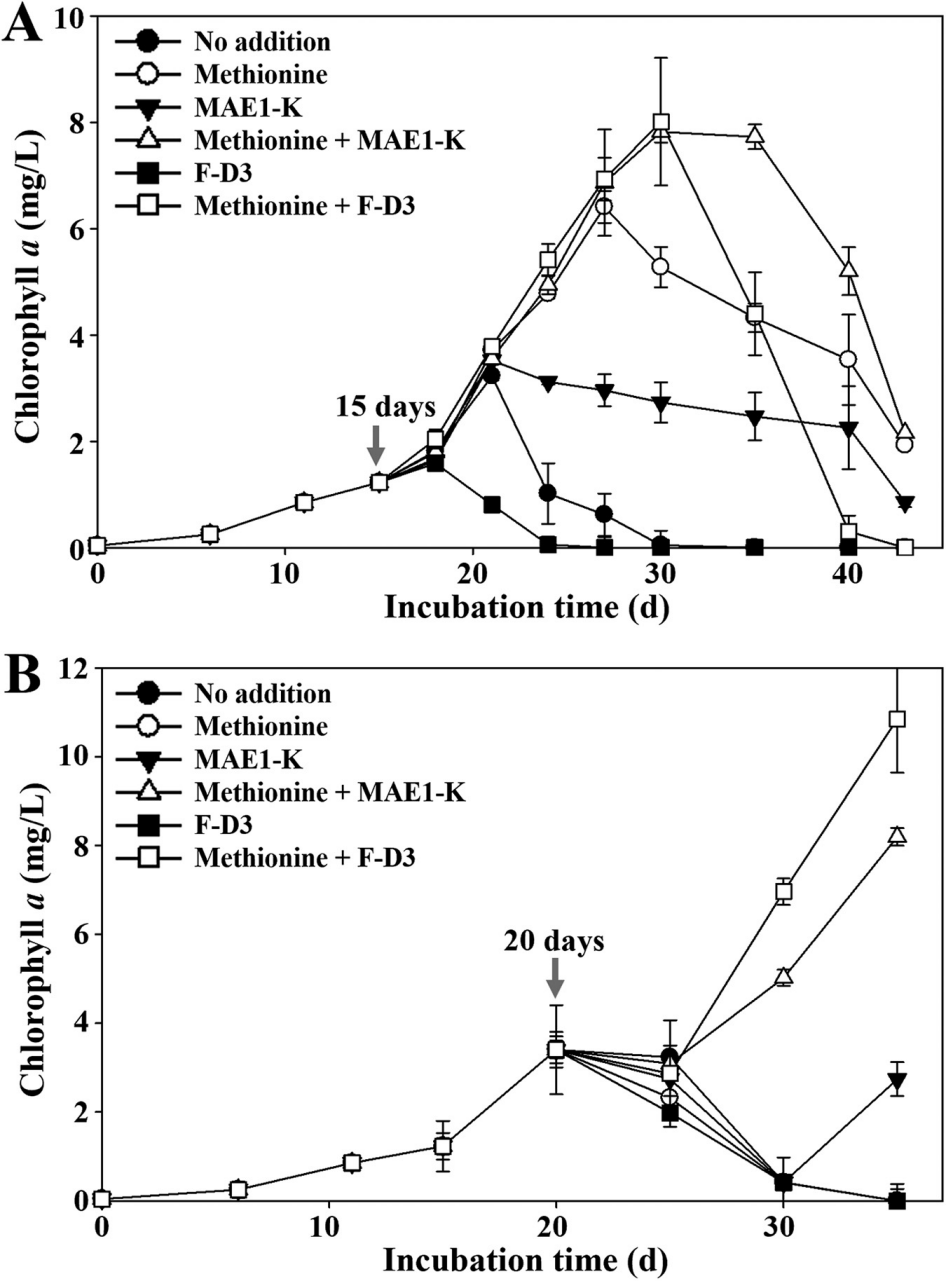
Effects of methionine on the growth of NIES-298 cultures. Methionine (0.5 mM), together with the inoculation of wild-type MAE1-K or mutant F-D3, was supplemented into the NIES-298 cultures of the middle (15 d, A) and late (20 d, B) exponential growth phases.

From the promoted bleaching of NIES-298 culture by strain F-D3, unable to synthesize methionine, we hypothesized that methionine deficiency may induce the production of bleaching compounds to cause the bleaching of *M. aeruginosa*, and thus the bleaching of *M. aeruginosa* can be delayed by the addition of methionine. Therefore, methionine, together with and without the inoculation of wild type MAE1-K or mutant F-D3, was supplemented into the NIES-298 cultures at the middle (15 d) or end (20 d) of exponential growth phases. The single inoculation of strain F-D3 quickly prompted the bleaching of NIES-298 cultures, which was thought to be because strain F-D3 might cause the deficiency of methionine inducing the production of the bleaching compounds by *M. aeruginosa* NIES-298 (Fig. 7). Supplementation of methionine at the middle of the exponential growth phase delayed the bleaching of NIES-298 cultures regardless of the inoculation of wild-type MAE1-K or mutant F-D3 (Fig. 7A). Additionally, methionine supplementation was better at delaying the bleaching of NIES-298 cultures than the inoculation of strain MAE1-K. Methionine supplementation, together with the inoculation of strain MAE1-K, significantly delayed the bleaching of NIES-298 cultures and increased maximum growth compared to the single inoculation of strain MAE1-K. Particularly, methionine supplementation, together with the inoculation of mutant F-D3, also significantly delayed the bleaching of NIES-298 cultures, like wild-type MAE1-K, but the cultures were quickly bleached after reaching the maximum growth, like in the axenic NIES-298 cultures.

However, methionine supplementation at the end of the exponential growth phase (20 d) showed somewhat different results from those at the middle of the exponential growth phase (Fig. 7B). The single supplementation of methionine did not delay the bleaching of NIES-298 cultures, which suggests that bleaching compounds were already produced to the extent of causing the bleaching of NIES-298 cultures even at the end of the exponential growth phase. However, methionine supplementation, together with the inoculation of wild-type MAE1-K or mutant F-D3, had a rescue effect with a time lag from the bleaching of NIES-298 cultures, which was thought to be because it took time for the bleaching compounds produced during the exponential growth phase to be inactivated by wild-type MAE1-K or mutant F-D3. The inoculation of mutant F-D3 with methionine supplementation had a better effect than wild-type MAE1-K, which is thought to be due to the better growth of mutant F-D3 in NIES-298 culture (Fig. 6C). NIES-298 cultures with the single inoculation of wild-type MAE1-K were significantly bleached but eventually grew again after 30 d. Taken together, the deficiency of methionine promotes the self-bleaching process of *M. aeruginosa*, and a symbiotic bacterium, Pseudomonas sp. MAE1-K, can delay the bleaching process of *M. aeruginosa* via the inactivation of the bleaching compounds.

## DISCUSSION

We observed that an axenic *M. aeruginosa* culture, NIES-298, was quickly bleached after maximum growth, like the sudden collapses of cyanobacterial blooms. Such collapses of cyanobacterial blooms are frequently observed in natural environments. Once the onset of bloom collapses occurs, blooms disappear within a few days (21, 22). Bloom collapses are usually observed during the summer–fall transition in temperate regions, but they are also often observed in tropical regions (9). Therefore, it has been considered that the sudden collapses of cyanobacteria blooms may be caused by other factors, such as salinity, pH, and nutrient level, besides temperature (23–24). Cyanophages have been considered one of the likely causes of the sudden collapses of cyanobacterial blooms (25–30). Therefore, at first, we considered the switch from lysogeny to a lytic life cycle of cyanophages as a cause of quick bleaching and lysis of NIES-298 cultures. However, genomic analysis and various efforts to detect cyanophages exhibited that the quick bleaching of the NIES-298 cultures was not caused by cyanophages.

Programmed cell death has also been suggested as one of the important cell death processes in various cyanobacteria including *M. aeruginosa* (31, 32). Caspases have been reported to promote programmed cell death (33), and oxidative stress is known to induce caspase gene-expression (34, 35). However, the transcriptional expression of genes associated with oxidative stresses or caspases was not upregulated during the bleaching periods of NIES-298 cultures. Additionally, the bleaching of NIES-298 cultures was not delayed or rescued by the treatment of SOD, catalase, or glutathione. These results suggest that oxidative stress or programmed cell death does not cause bleaching of *M. aeruginosa*.

The loss of green color, called chlorosis, has been frequently reported in various nondiazotrophic cyanobacteria such as *Synechococcus*, *Phormidium tenue*, and *M. aeruginosa*, and the depletion of nutrients, particularly nitrogen limitation, has been considered as the main cause (8, 36–40). The cyanobacteria in nitrogen-limited conditions switch to a bleaching state with low photosynthesis, called chlorosis, through the reduction of phycobiliprotein and chlorophyll-*a* content for their long-term survival. It was reported that the *nblA* gene encoding NblA, a Clp protease-associated adapter protein mediating the degradation of phycobilisomes, was induced by nitrate depletion, which resulted in the chlorosis of *Synechococcus* (41). This study showed that the putative *nblA* gene, identified from the genome of *M. aeruginosa* NIES-298, was slightly upregulated during the bleaching period. However, the nitrogen source (nitrate) was not depleted during the entire growth period of NIES-298 cultures. The bleaching of NIES-298 cultures changing to white is also clearly differentiated from the chlorosis process of *Synechococcus* showing a color change to yellowish-green. Cells of cyanobacteria in chlorosis can be revived into normal metabolic cells in nutrient-replete conditions (39), but the bleaching of *M. aeruginosa* NIES-298 cultures eventually leads to lysis and cell death. These results suggest that the bleaching of *M. aeruginosa* is fundamentally different from the nutrient depletion-induced chlorosis.

It has been reported that in various cyanobacteria including *Anabaena*, *Prochlorococcus*, *Synechococcus*, and *Microcystis*, symbiotic heterotrophic bacteria benefit the proliferation of cyanobacteria by providing nutrients, increasing trace metal availability, alleviating oxidative stresses, and eliminating external toxic compounds (6, 42, 43). Additionally, long-term survival of cyanobacteria in nutrient-starved conditions relies on symbiotic heterotrophic bacteria (8, 40, 44). For example, a symbiotic *Roseobacter* strain mineralizes organic matter produced by photosynthetic *Synechococcus*, and *Synechococcus* utilizes the degraded compounds again in nutrient-depleted conditions (44). *Prochlorococcus* has chlorotic cells, not resting stages, unable to regrow in the oligotrophic marine environments, and heterotrophic bacteria enables *Prochlorococcus* to survive long-term stress without resting stages (8). Our study also showed that *M. aeruginosa* has a bleaching process that makes it unable to regrow, and the long-term proliferation of *M. aeruginosa* may be highly dependent on some symbiotic heterotrophic microbes. A heterotrophic Pseudomonas sp. MAE1-K isolated from the xenic KW culture of *M. aeruginosa* enables axenic NIES-298 cultures to survive without bleaching through the inactivation of the bleaching compounds produced by *M. aeruginosa* (Fig. 5).

The screening for mutants of strain MAE1-K that lost the ability to delay the bleaching of NIES-298 cultures from a Tn5 transposon library failed, but we found that methionine deficiency may be closely associated with the bleaching process of *M. aeruginosa*. The inoculation of a *metZ* mutant (F-D3) probably leading to methionine deficiency promoted the bleaching process of NIES-298 cultures, while methionine supplementation significantly delayed the bleaching of NIES-298 cultures (Fig. 6 and 7). Methionine is an essential amino acid for protein synthesis, and *S*-adenosylmethionine, a methionine metabolite, is an indispensable methyl donor for various biochemical reactions including chlorophyll biosynthesis and many other methylation reactions (e.g., DNA and RNA methylation and posttranscriptional modification) (45). Therefore, methionine deficiency may be fatal for growth or survival of *M. aeruginosa*, which may lead to the production of bleaching compounds causing the self-bleaching process of *M. aeruginosa*. *M. aeruginosa* NIES-298 can synthesize methionine and thus it can grow in BG-11 media without methionine. A significant portion of methionine synthesized by *M. aeruginosa* may be secreted into the culture solution, and *M. aeruginosa* may reuse it through uptake (46). In this study, the growth of mutant F-D3 was observed in filtered NIES-298 cultures without the supplementation of methionine, representing the presence of methionine. Therefore, if a *metZ* mutant, unable to synthesize methionine, intercepts the secreted methionine for growth, *M. aeruginosa* cannot obtain enough methionine for growth, representing methionine deficiency.

Dagnino and colleagues reported that the bleached cultures of *M. aeruginosa* PCC 7806 induced bleaching of the exponentially growing cells in nutrient-replete conditions (36), and the same results were observed from *M. aeruginosa* NIES-298 of our study. Additionally, the bleaching process was also observed from other xenic *M. aeruginosa* cultures, KW, FBC0000003, and NICRCY0000000452, although a longer incubation time was required. These results suggest that bleaching by *M. aeruginosa* self-produced bleaching compounds may be a common cell death process in *M. aeruginosa*. Due to the genetic regulation of the process and the regeneration ability in nutrient-replete conditions, chlorosis is considered an adaptation strategy of cyanobacteria that seeks to maintain long-term survival through very low metabolic activity in nutrient-deficient environments (47). Additionally, programmed cell death by genetic regulation also is accepted as a strategy of cyanobacteria for survival under stress conditions (34). However, unlike chlorosis or programmed cell death, genes that may be associated with chlorosis or programmed cell death were not induced during the bleaching of *M. aeruginosa*, and cell viability also was not maintained after the bleaching process, which suggests that the bleaching process of *M. aeruginosa* may not be a survival or adaption strategy of *M. aeruginosa* to respond to environmental stresses, unlike chlorosis or programmed cell death. Therefore, further research is needed on why and how *M. aeruginosa* has a self-bleaching process that does not have cell viability. Additionally, it is necessary to study how methionine deficiency induces the bleaching process of *M. aeruginosa* and how a symbiotic bacterium, strain MAE1-K, inactivates the bleaching compounds to delay the bleaching process of *M. aeruginosa*. Nevertheless, this study provides a better understanding of the physiological and ecological features of *M. aeruginosa* and the interactions between *M. aeruginosa* and symbiotic bacteria, which will contribute to developing strategies to control cyanobacterial blooms.

## MATERIALS AND METHODS

### Microcystis aeruginosa strains and culture conditions.

Three xenic *M. aeruginosa* cultures, KW, NIBRCY000000452, and FBC000003, were provided by the Korea Research Institute of Bioscience and Biotechnology, National Institute of Biological Resources (Korea), and Nakdonggang National Institute of Biological Resources (Korea), respectively, and an axenic *M. aeruginosa* culture, NIES-298, was purchased from the National Institute for Environmental Studies (Japan). All *M. aeruginosa* cultures were grown in sterilized BG-11 broth at 25°C under 40 μmol photons m^−2^·s^−1^ with a 12 h/12 h light/dark cycle (48). *M. aeruginosa* cultures were subcultured by transferring 2% cultures of exponential growth phase into 50 mL fresh BG-11 broth in a 125-mL air-filtered cell culture flask (SPL Life Science, Korea). Here, their growth was assessed by measuring chlorophyll-*a* concentration according to an acetone extraction method described previously, except where indicated otherwise (49). *M. aeruginosa* cell morphologies were observed using a differential interference contrast microscope (DICM; Carl Zeiss Axio Scope.A1, Germany) and a transmission electron microscope (TEM; JEM-1010, JEOL, Japan).

### Effects of various factors on the growth of NIES-298 culture.

The genome of strain NIES-298 was completely sequenced by a combination of PacBio RS II SMRT sequencing with a 10-kb library and Illumina HiSeq 4000 paired-end sequencing (151 bp) at Macrogen (Korea), according to the procedure described previously (50). Cyanophage genes in the genome of strain NIES-298 were identified using PHASTER (51). Detection of cyanophages present in NIES-298 culture was done according to a previously described procedure (52). Viral cell lysis in NIES-298 culture was evaluated by determining the formation of viral plaques after spreading the NIES-298 cultures on BG-11 agarose (0.8%, wt/vol). Nitrate concentration in the NIES-298 cultures was measured according to a previously described method (53). The effects of superoxide dismutase (SOD, 200 U/mL), catalase (200 U/mL), and glutathione (0.5 mM) on the growth of NIES-298 cultures were assessed by treating them to the NIES-298 cultures of exponential or death phases. To investigate the effects of the cultured solution of NIES-298 on the cell growth of NIES-298, NIES-298 cultures of the exponential growth (18 d in Fig. 1) and death (23 d in Fig. 1) phases were filtered using a 0.45 μm filter. Exponentially growing NIES-298 cultures (2%) were inoculated into BG-11 broth supplemented with the filtered NIES-298 cultured solution and their growths were evaluated.

### Transcriptomic analysis.

The total RNA of axenic NIES-298 cultures at the middle exponential, late exponential, and early death phases was extracted using an RNeasy minikit (Qiagen, Germany) and sequenced using an Illumina HiSeq 4000 platform at Macrogen (Korea). Low-quality sequencing reads were removed using Sickle (https://github.com/najoshi/sickle), and high-quality mRNA reads were mapped onto the genome of strain NIES-298 using Burrows-Wheeler Aligner (BWA; http://bio-bwa.sourceforge.net), as described previously (54). Gene expression levels were calculated as reads per kilobase of gene per million mapped reads (RPKM).

### Isolation of bacteria from KW culture and its effects on the growth of NIES-298 culture.

Bacterial strains were isolated from a xenic KW culture, according to a procedure described previously with some modifications (55). Briefly, the xenic KW culture was mechanically homogenized, serially diluted, and spread on R2A agar (BD, USA). After 3 d of incubation at 25°C, 16S rRNA genes from 50 randomly selected colonies were amplified and double-digested with a mixture of HaeIII and HhaI. Representative PCR products of different fragment patterns were sequenced, and their sequences were compared with those of all validated type strains using the Nucleotide Similarity Search program in the EzBioCloud (56). To evaluate the effects of the bacterial isolates on the growth of NIES-298 culture, bacterial isolates were cultured in R2A broth, washed using BG-11 broth, and inoculated into fresh NIES-298 cultures to a final concentration of 10^7^ bacterial cells/mL.

The inactivation test for the bleaching property of bleached culture by strain MAE1-K, bleached NIES-298 culture obtained from the end of the death phase was filtered using a 0.45 μm filter (Millipore, USA). Strain MAE1-K was cultured in R2A broth, washed with BG-11 broth, and inoculated into the filtered bleached culture (approximately 10^6^ cells/mL). After 24 h of aerobic incubation at 25°C, bleached culture treated with strain MAE1-K was filtered again using a 0.45 μm filter and used for the growth tests.

### Construction and screening of a Tn5 transposon mutant library of strain MAE1-K.

The genome of strain MAE1-K was completely sequenced using the same procedure as that used for the genome sequencing of strain NIES-298. A Tn5 transposon mutant library of strain MAE1-K was constructed using the EZ-Tn5 <TET-1> Insertion Kit (Lucigen, USA), according to the manufacturer’s instructions. For screening of knockout mutants of strain MAE1-K that lost the ability to delay bleaching of NIES-298 cultures, all Tn5 transposon mutants of strain MAE1-K were cultivated in R2A broth containing tetracycline (10 μg/mL) at 30°C, washed using BG-11 broth, and inoculated into 400 μL axenic NIES-298 cultures of the middle exponential growth phase in 96-well plates (approximately 10^7^ cells/mL). The 96-well plates were incubated at 25°C under 40 μmol photons m^−2^·s^−1^ with a 12 h/12 h light/dark cycle, and the growth of NIES-298 cultures was assessed by measuring optical density (680 nm).

The transposon insertion site of a knockout mutant, which completely lost the ability of strain MAE1-K to delay bleaching of NIES-298 cultures, was identified through a two-round PCR approach (57). Briefly, a sequence adjacent to the transposon insertion site was amplified using a transposon-specific primer TET-1 FP-A (5′-GCG ACG CGA GGC TGG ATG G-3′) and an arbitrary primer ARB1D (5′-GGC CAG GCC TGC AGA TGA TGN NNN NNN GTA T-3′), followed by a second amplification using a nested transposon-specific primer TET-1 FP-1 (5′-CGC ATG ATC CTC TAG AGT-3′) and a primer corresponding to a nonrandom portion of the arbitrary primer ARB2A (5′-GGC CAG GCC TGC AGA TGA TG-3′). The final PCR product was sequenced, and transposon insertion site was identified by comparing the sequence with the genome sequence of strain MAE1-K. The growth of mutant F-D3 in R2A broth, BG-11 broth supplemented with glucose (0.1%, wt/vol) and methionine (1 mM), and filtered bleached culture in the death phase was evaluated. The inactivation test for the bleaching property of the bleached culture by mutant F-D3 was conducted in wild-type MAE1-K as described above.

### Effects of methionine on the bleaching of NIES-298 cultures.

To investigate the effects of methionine on the bleaching of NIES-298 cultures, methionine was added to the NIES-298 cultures in the middle and late exponential growth phases (0.5 mM). Wild type MAE1-K and mutant F-D3 were cultivated in R2A broth, washed using BG-11 broth, and inoculated into NIES-298 cultures (10^7^ cells/mL).

### Data availability.

Genomes of *M. aeruginosa* NIES-298 and Pseudomonas sp. MAE1-K are deposited in the NCBI GenBank database under the accession numbers CP046058-9 and CP023641, respectively. Raw data of RNA-seq performed at the mid-exponential, late exponential, and early death phases of *M. aeruginosa* NIES-298 are available under accession number of BioProject PRJNA802958 and SRA SRR17982185–7.
