# Supplementary material for: The Self-Bleaching Process of Microcystis aeruginosa is Delayed by a Symbiotic Bacterium Pseudomonas sp. MAE1-K and Promoted by Methionine Deficiency
Source: Microbiol Spectr. 2022 Jun 30;10(4):e01814-22. doi: 10.1128/spectrum.01814-22 (PMC9430746; doi:10.1128/spectrum.01814-22)
Supplement: Supplemental file 1 — Supplemental material. Download spectrum.01814-22-s0001.pdf, PDF file, 0.6 MB [file spectrum.01814-22-s0001.pdf]

Supplemental Material

Fig. S1. Differential interference-contrast microscopic cell images of the xenic (KW) and axenic (NIES-298) cultures of *M. aeruginosa* during growth. The xenic KW cells stayed in healthy and green states even after 28 d, whereas the axenic NIES-298 cells were mostly bleached and lysed after 23 d.

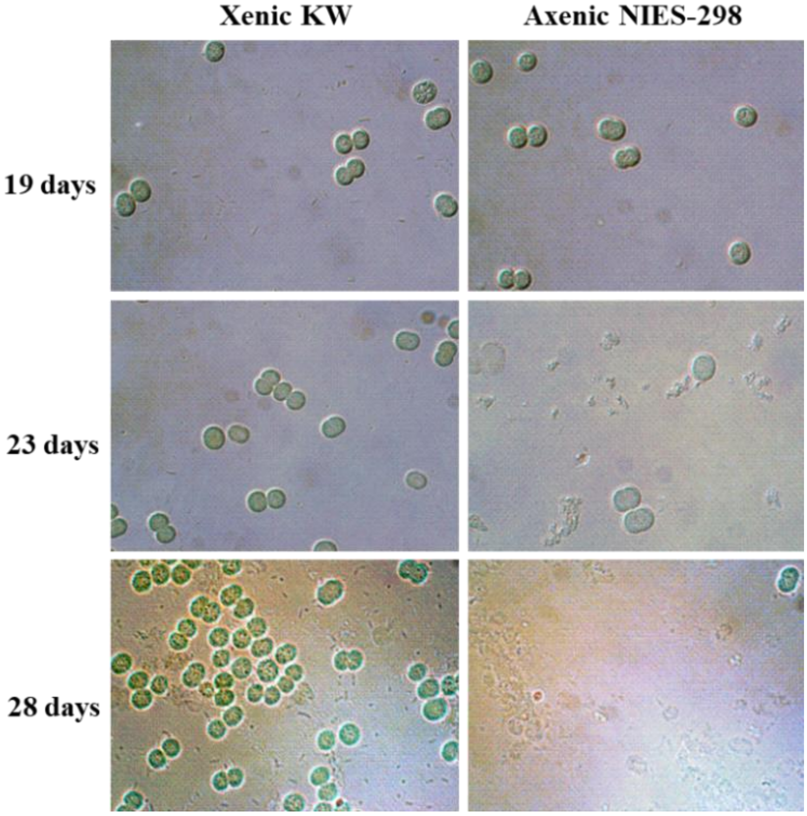

Fig. S2. Circular maps (A) of the genome of *M. aeruginosa* NIES-298 consisting of one chromosome (CP046058) and one plasmid (CP046059). The circular maps of the genome were generated using a web-based CGview program (http://cgview.ca/) (A). Many putative cyanophage-associated genes were identified, but they were separately distributed into two distant regions of the chromosome. The putative functions and physical maps of the predicted cyanophage genes were indicated (B).

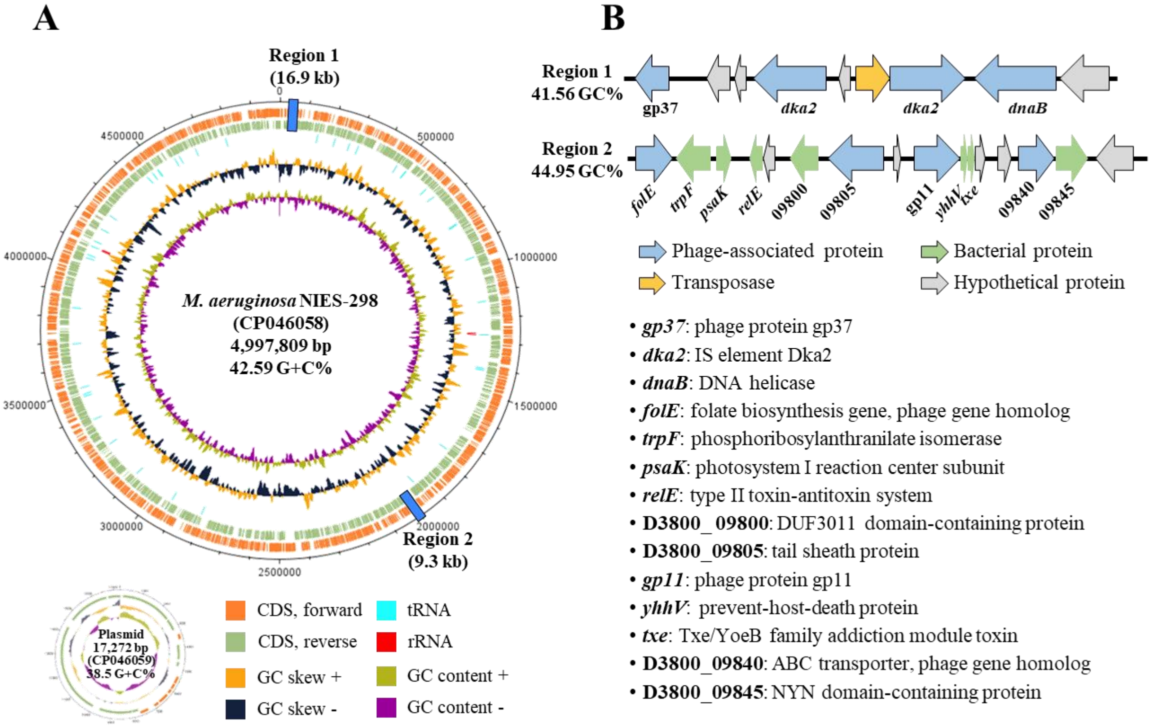

Fig. S3. Viral plaque formation test of NIES-298 culture. NIES-298 culture was streaked on BG11 agarose (0.8%, w/v) and viral plaque formation was checked, which showed that NIES-298 cells were bleached without viral plaque formation.

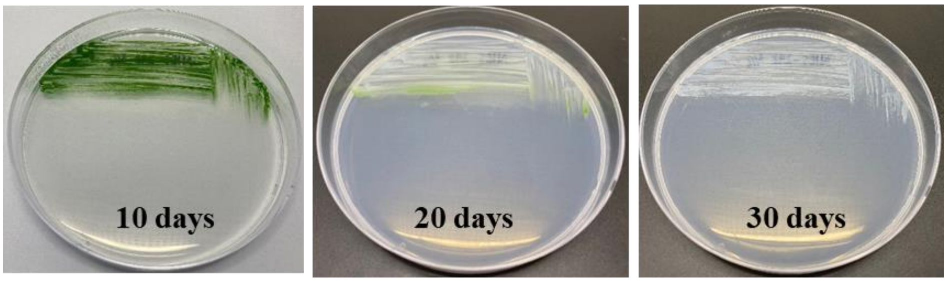

Fig. S4. Nitrate concentrations of BG-11 broth during the growth of NIES-298 culture.

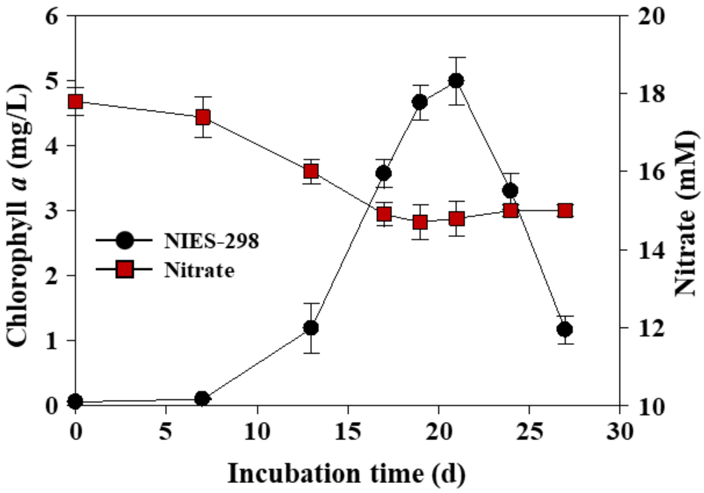

Fig. S5. A circular map representing the genome of *Pseudomonas* sp. MAE1-K (GenBank acc. no. CP023641). The circular map was generated using the web-based CGview program (http://cgview.ca/).

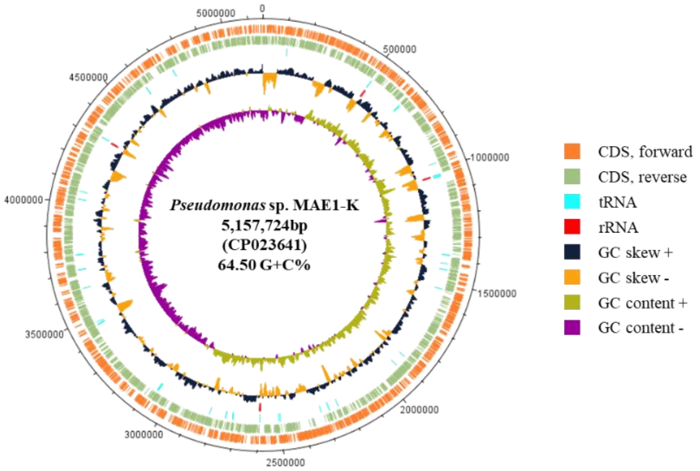

Table S1. Transcriptional gene expression of *M. aeruginosa* NIES-298 cells at different growth phases, mid-exponential (ME) growth, late-exponential (ME) growth, and early death (ED) phases. Differentially expressed genes (|Log_2fold-changes|>1) at the ED phase compared to the ME or LE growth phases are listed. Gene expression levels were indicated as read numbers per kilobase of gene per million mapped reads (RPKM). Genes coding hypothetical proteins or with low expression levels (RPKM<50) were not listed. The Illumina sequencing data of the transcriptomes derived in this study are publicly available in the NCBI Sequence Read Archive (SRA) under the accession numbers of SRR17982185–7 (NCBI BioProject accession number PRJNA802958).

Table S2. Identification and abundances of bacterial strains isolated from xenic KW culture of *M. aeruginosa.*

Strain	Closest type strain	Similarity (%)[a]	Abundance (%)[b]
MAE1-K	*Pseudomonas mendocina* NBRC 14162^T	99.73	62
MAE2-X	*Rhizobium rosettiformans* W3^T	99.78	24
MAE2-B	*Rhizobium radiobacter* ATCC 19358^T	99.36	10
MAE2-J	*Rhodococcus jialingiae* JCM 15477^T	100	2
MAE2-T	*Bosea vestrisii* 34635^T	99.72	2

[a] 16S rRNA gene sequence similarity
[b] A total of 50 colonies were analyzed.
